# Differentiation of local tumor recurrence from radiation-induced changes after stereotactic radiosurgery for treatment of brain metastasis: case report and review of the literature

**DOI:** 10.1186/1748-717X-8-52

**Published:** 2013-03-06

**Authors:** Philipp Kickingereder, Franziska Dorn, Tobias Blau, Matthias Schmidt, Martin Kocher, Norbert Galldiks, Maximilian I Ruge

**Affiliations:** 1 Department for Stereotaxy and Functional Neurosurgery, University of Cologne, Kerpener Str. 62, 50937, Cologne, Germany; 2 Department of Radiology, University of Cologne, Cologne, Germany; 3 Department of Neuropathology, University of Cologne, Cologne, Germany; 4 Department of Nuclear Medicine, University of Cologne, Cologne, Germany; 5 Department of Radiation Oncology, University of Cologne, Cologne, Germany; 6 Department of Neurology, University of Cologne, Cologne, Germany; 7 Institute of Neuroscience and Medicine, Research Center Jülich, Jülich, Germany

**Keywords:** Brain metastases, Radiation-induced changes, Tumor recurrence, Stereotactic-guided biopsy, PET, Advanced MRI techniques

## Abstract

**Background:**

Structural follow-up magnetic resonance imaging (MRI) after stereotactic radiosurgery (SRS) for brain metastases frequently displays local changes in the area of applied irradiation, which are often difficult to interpret (e.g., local tumor recurrence, radiation-induced changes). The use of stereotactic biopsy for histological assessment of these changes has a high diagnostic accuracy and can be considered as method of choice. In order to solve this relevant clinical problem non-invasively, advanced MRI techniques and amino acid positron-emission-tomography (PET) are increasingly used.

**Case presentation:**

We report the long-term follow-up of a patient who had been treated with linear accelerator based SRS for cerebral metastases of a lung cancer. Fifty-eight months after SRS, the differentiation of local recurrent brain metastasis from radiation-induced changes using structural MRI was difficult. For further differentiation, perfusion-weighted MRI (PWI), proton magnetic resonance spectroscopy (MRS), and ^11^C-methyl-L-methionine (MET) PET was performed. Due to artifacts and technical limitations, PWI MRI and MRS findings were not conclusive. In contrast, MET PET findings were suggestive for radiation-induced changes. Finally, a stereotactic biopsy for histological assessment of these changes demonstrated clearly a radiation-induced necrosis and the absence of vital tumor.

**Conclusion:**

The use of stereotactic biopsy for histological assessment of indistinguishable lesions on structural MRI after SRS for treatment of brain metastasis represents a highly reliable method to differentiate local tumor recurrence from radiation-induced changes. In this field, results of studies with both advanced MRI techniques and amino acid PET suggest encouraging results. However, artifacts and technical limitations (e.g., lesion size) are still a problem and comparative studies are needed to investigate the relationship, diagnostic performance, and complementary character of advanced MRI techniques and amino acid PET.

## Background

Early detection of brain metastasis provided by advanced screening programs as well as improvements of treatment in general oncology is positively influencing the course of the disease and overall survival of patients with brain metastases. To date, the use of stereotactic radiosurgery (SRS) is a widely accepted treatment option for (singular) cerebral metastasis [1-3]. As a consequence, the appearance of local signal changes on follow-up MRI in the irradiated region is observed in up to 22% of cases after SRS [4]. The interpretation of findings of structural magnetic resonance imaging (MRI) at follow-up after treatment with SRS (i.e., changing patterns of contrast enhancement and alterations of T2-/FLAIR-weighted images) is difficult or even impossible in order to differentiate local tumor recurrence from radiation-induced changes [5-8]. Furthermore, little experience is reported whether and when these changes occur in patients during long-term follow-up.

We present a follow-up of more than 5 years of a patient after treatment of cerebral metastases using SRS. During follow-up, the assignment of structural MRI changes to local tumor recurrence or radiation-induced changes was difficult. Regarding this clinically relevant issue, we discuss noninvasive advanced imaging methods based on MRI (i.e., perfusion-weighted MRI (PWI), proton magnetic resonance spectroscopy (MRS), and positron-emission-tomography (PET)).

## Case presentation

**Figure 1:**
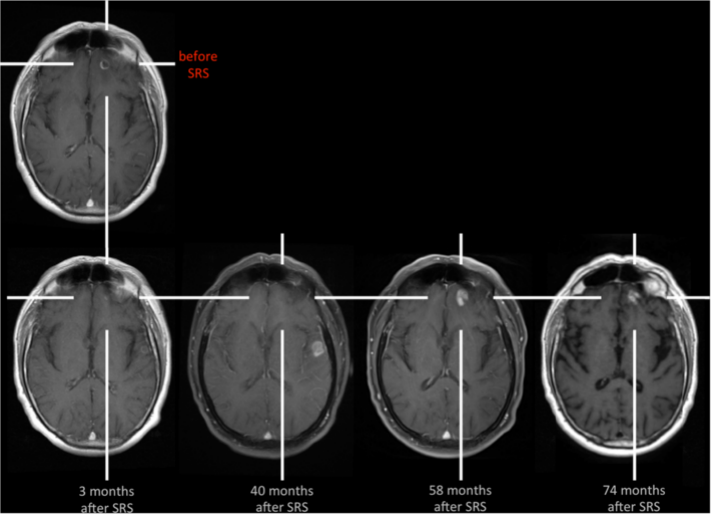
**Contrast-enhanced MR images during the course of the disease.** Fifty-eight months after LINAC SRS of the left frontal metastasis, contrast enhancement recurs within the area of applied irradiation. A differentiation of local tumor recurrence from radiation-induced changes is not possible.

In November 2005, in a 58-year-old male patient an adenocarcinoma of the lung was diagnosed and he underwent multimodal treatment including resection of the upper pulmonary lobe and adjuvant chemotherapy. Nine months later (July 2006), a follow-up MRI revealed a contrast-enhancing solitary lesion in the left frontal brain, highly suggestive for a brain metastasis originating from adenocarcinoma of the lung (Figure 1). The lesion was treated by linear accelerator (LINAC) based SRS (tumor volume, 0.6 ml; surface dose, 20 Gy; isodose level, 65%) [9,10]. Follow-up MRI after 3 months (Figure 1) showed a decrease of contrast enhancement > 50% of the treated lesion (partial response according to the Macdonald criteria [11]) as well as a new enhancing lesion also suggestive for a metastasis in the left temporal lobe (Figure 1), which was also treated with LINAC based SRS (tumor volume, 1.2 ml; surface dose, 20 Gy; isodose level, 65%). The subsequent MRI performed in December 2006 showed complete remission of both metastases (images not shown).

Thirty-seven months after SRS of the left temporal metastasis, a new contrast-enhancing lesion with perifocal edema was detected in the area of applied irradiation (Figure 1). Due to further progression of size in the follow-up MRI one month later (tumor volume, 2.8 ml), the lesion was highly suspicious for local tumor recurrence. In order to differentiate local tumor recurrence from radiation-induced changes, a stereotactic biopsy was performed. Histological assessment revealed a radiation-induced necrosis. Tumor cells were completely absent. The diagnosis of a radiation-induced necrosis was additionally confirmed by the clinical and radiological course. In follow-up MR imaging, the contrast enhancement vanished completely (Figure 1).

**Figure 2:**
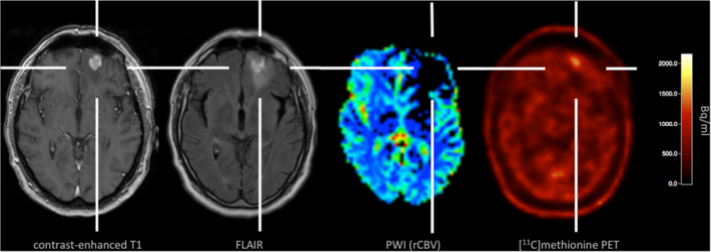
**Multimodal imaging with structural MRI (contrast-enhanced T1- and FLAIR-weighted sequence), PWI MRI, and MET PET at the time point of suspected tumor recurrence.** PWI MRI shows a perfusion deficit, areas with increased relative cerebral blood volume (rCBV) are not present. In contrast, MET PET is suggestive for radiation-induced changes (mean tumor-to-brain ratio, 1.40).

Fifty-eight months after SRS of the left frontal metastasis, we observed a recurrence of contrast enhancement (tumor volume, 1.8 ml) within the area of applied irradiation accompanied by a hyperintensity in the FLAIR-weighted MRI (Figure 1, 2). For further evaluation of this lesion, PWI MRI, MRS, and ^11^C-methyl-L-methionine (MET) PET was performed.

**Figure 3:**
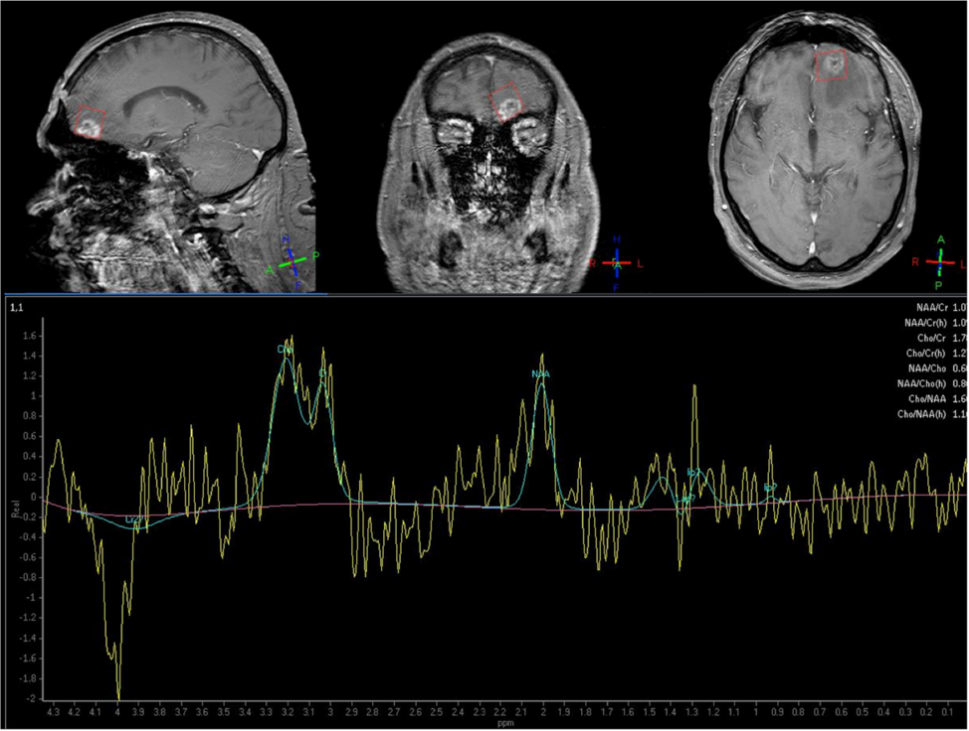
Spectroscopic profile of the left frontal lesion (red voxel), altered by artifacts.

**Figure 4:**
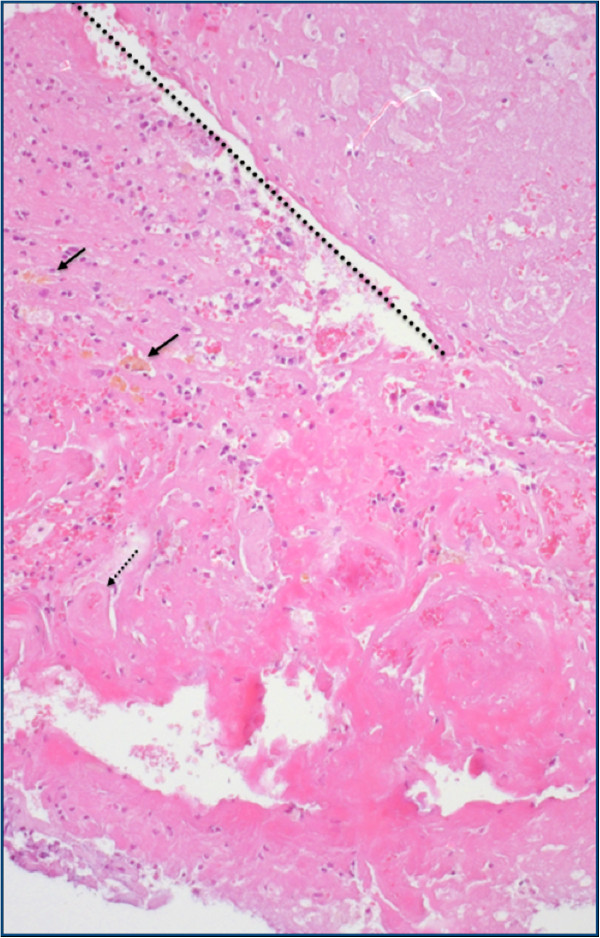
**Histology of left frontal tissue, obtained by stereotactic biopsy.** Hyalinized radiation-induced necrosis, H&E staining, original magnification x100. Within the necrosis, blood vessels with thickened walls and obturated lumen (dotted arrow). On the left side, vital brain parenchyma with hemosiderin deposits (arrows).

In detail, PWI MRI revealed a perfusion deficit with low mean transit time and low relative cerebral blood volume (rCBV), which was corresponding to the signal alterations on the FLAIR-weighted image (Figure 2). Areas with increased rCBV, however, could not be clearly identified within the perfusion deficit. Furthermore, diagnostic information of MRS was heavily influenced by artifacts and was therefore inconclusive (Figure 3). It should be noted that most probably due to the patient’s unusual large frontal sinus adjacent to the left frontal lesion, the diagnostic value of PWI MRI, and MRS might have been negatively influenced. MET PET imaging of the suspicious left frontal lesion revealed a mean tumor-to-brain ratio of 1.40 (normalized against the gray matter of the unaffected right frontal cortex) (Figure 2). It has been previously demonstrated that a mean threshold of at least 1.41 or 1.42 has the best diagnostic accuracy for the identification of recurrent brain metastasis [12,13]. Accordingly, the tumor-to-brain ratio of 1.40 was suggestive for radiation-induced necrosis. Nevertheless, the ratio of 1.40 was very close to the thresholds for diagnosis of recurrent metastasis (1.41 and 1.42, respectively) and MR imaging findings were not helpful. We therefore decided to perform a stereotactic biopsy for histological evaluation of the suspicious left frontal lesion. According to our previously reported protocol, a stereotactic ^125^I brachytherapy (SBT) after intraoperative verification of tumor within the same surgical procedure was planned [6,14]. Intraoperatively, histological findings were consistent with radiation necrosis (Figure 4). Thus, SBT was not performed.

Follow-up MRI after 74 months showed a decrease of contrast enhancement (Figure 1). During the complete course of the disease, the patient was asymptomatic and had no neurological deficit. Furthermore, there were no clinical signs for systemic disease progression.

## Conclusions

To date, contrast-enhanced structural MRI is the method of choice for follow-up of brain metastasis after treatment with SRS. However, in many patients, the differentiation of local recurrent brain metastasis from radiation-induced changes after SRS using contrast-enhanced MRI is difficult [15]. This problem necessitates alternative diagnostic methods for the follow-up and management of patients with recurrent brain metastases. Various imaging techniques such as PET, single-photon emission computed tomography (SPECT), MRS, and PWI have been used for differentiation of local tumor recurrence from radiation-induced changes (Table 1).

**Table 1:** Diagnostic value of several imaging techniques in differentiating tumor recurrence from radiation necrosis after SRS for brain metastases

**Study**	**Imaging modality**	**Number of patients (metastasis)**	**Treatment (n)**	**Interval SRS – Imaging (mo)**	**Number of recurrent metastasis / radiation-induced changes**	**Confirmation of diagnosis (n)**		**Sensitivity**	**Specificity**
						**Histo**	**FU**		
Serizawa et al., 2005 [16]	^201^Thallium SPECT	70 (72)	SRS	7	30/42	10	62	90%	91%
Tsuyuguchi et al., 2003 [12]	MET PET	21 (21)	SRS	12	9/12	11	10	78%	100%
Terakawa et al., 2008 [13]	MET PET	51 (56)	SRS (47), EBI (4)	17	24/32	56	0	79%	75%
Galldiks et al., 2012 [17]	FET PET	31 (40)	SRS (16), SRS+WBRT (15)	12	19/21	11	29	95%	91%
Horky et al., 2010 [18]	Dual phase FDG PET	25 (27)	SRS/EBI (22), CTx (3)	18,15	n.r.	16	11	95%	100%
Chao et al., 2001 [19]	FDG PET	32 (36)	SRS	6	18/18	n.r.	n.r.	65%	80%
	FDG PET + MRI	32 (36)	SRS	6	18/18	n.r.	n.r.	86%	80%
Belohlavek et al., 2003 [20]	FDG PET	25 (57)	SRS	8	8/49	3	54	75%	94%
Chernov et al., 2005 [21]	FDG PET	9 (9)	SRS	11	4/5	5	4	50%	80%
	Single voxel MRS	9 (9)	SRS	11	4/5	5	4	50%	100%
	Multi voxel MRS	9 (9)	SRS	11	4/5	5	4	100%	100%
Kimura et al., 2003 [22]	Single voxel MRS	36 (43)	SRS	6-12	0/6	5	1	100%*									
Barajas et al., 2009 [23]	PWI	27 (30)	SRS	10	20/10	22	8	96%	100%								
Hoefnagels et al., 2009 [4]	PWI	31 (34)	SRS	8/9	20/14	11	23	70%	100%								
Mitsuya et al., 2010 [24]	PWI	27 (28)	SRS	12	7/21	2	26	100%	95%								
Truong et al., 2006 [25]	PWI	12**	SRS	9	10/2	12	0	PPV 80%									
	Multi voxel MRS	9***	SRS	9	7/2	9	0	PPV 83%									

*Radiation necrosis was correctly diagnosed in all cases; **3 patients were excluded due to susceptibility artifacts; ***2 patients were excluded.

*CTx* = chemotherapy; *PWI* = perfusion weighted imaging; *EBI* = external beam irradiation; *FDG* = 2-deoxy-[^18^F]fluoro-D-glucose; *FET* = O-(2-^18^F-Fluoroethyl)-L-tyrosine; *FLAIR* = fluid attenuated inversion recovery; *FU* = confirmation of diagnosis by (serial) follow-up MRI; *Histo* = diagnosis proven by histological confirmation; *MET* = ^11^C-methyl-L-methionine; *MRS* = magnetic resonance spectroscopy; *n.r.* = not reported; *PPV* = positive predictive value for tumor recurrence; *SPECT* = single photon emission computed tomography; *SRS* = stereotactic radiosurgery; *WBRT* = whole brain radiation therapy.

PET using 2-deoxy-[^18^F]fluoro-D-glucose (FDG) [26] has been considered for the evaluation of brain metastasis, but the high physiologic glucose consumption of the brain and the variable glucose uptake of metastatic brain lesions limit its use. For example, in a study of 48 patients with lung cancer and brain metastasis, 33% of the brain lesions could not be clearly detected by FDG PET, although all primary lung lesions were hypermetabolic [27]. Furthermore, another limitation is a small lesion size [28]. Another study demonstrated that after SRS, FDG PET is not sensitive enough to differentiate brain metastases from radiation necrosis [20]. Additionally, the diagnostic accuracy of FDG PET may be negatively influenced since it could be observed that FDG uptake is also seen in inflammatory changes [29]. A recent study indicated that dual-phase imaging may improve the diagnostic accuracy of FDG PET for the differentiation of recurrent brain metastasis from radiation necrosis [18]. A relevant limitation of that approach is the long time interval between PET scans (range, 2–5.7 h).

Amino acid tracers are particularly useful for PET in Neurooncology because of a high amino acid uptake in tumor tissue both in metastasis and gliomas [30] together with low uptake in normal brain tissue, resulting in an enhanced tumor-to-background contrast. Previously, it has been shown that PET using MET may be effective in differentiating recurrent metastatic brain tumor from radiation-induced changes (at a threshold of 1.42, mean tumor-to-brain ratio) with a sensitivity of 78% and a specificity of 100%, respectively [12]. In a subsequent study [13], these findings could be confirmed at a similar threshold (1.41, mean tumor-to-brain ratio), at least in part, with similar sensitivity (79%) but with lower specificity (75%). Following these thresholds (1.41 and 1.42, respectively), the mean tumor-to-brain ratio of 1.40 of the left frontal lesion in our patient was suggestive for radiation-induced changes. However, this ratio was very close to the reported thresholds for diagnosis of recurrent metastasis and might therefore cause several concerns regarding the correct diagnosis. For example, the calculated mean tumor-to-brain ratio may slightly vary depending on the choice of the background region-of-interest (ROI). It should be noted that the use of MET remains restricted to centers with an on-site cyclotron because of the short half-life of the ^11^C isotope (20 min). In contrast, amino acids labeled with ^18^F (half-life, 110 min) such as O-(2-^18^F-fluoroethyl)-L-tyrosine (FET) allow a more widespread use. In a recent study it could be demonstrated that the combination of the mean tumor-to-brain ratio and FET kinetics helps to differentiate recurrent brain metastasis tumor from radiation-induced changes with a high diagnostic accuracy [17].

A study with ^201^Thallium SPECT provided the correct diagnosis of recurrent brain metastasis with a sensitivity of 90% and specificity of 92%, respectively [16]. However, the spatial resolution of SPECT is considerably lower than that of PET, which limits the use of this technique in clinical practice.

Beyond structural MR imaging, advanced MRI techniques such as PWI and MRS can be used successfully in order to differentiate recurrent brain metastasis from radiation-induced changes. Especially PWI can be easily incorporated into daily practice since the acquisition time is short (less than 10 minutes) [31]. In this context, the diagnostic value of PWI and MRS has been investigated by several studies [4,23-25,32]. In these studies, the sensitivity of PWI MRI ranged from of 70–100% and the specificity from 95–100%, respectively. Chernov and colleagues reported a sensitivity and specificity of 100% using MRS in pilot series of 9 patients [21]. However, it should be noted that these promising results of studies with advanced MRI techniques are mostly based on a limited number of patients investigated predominantly in a retrospective setting, without histological confirmation of the diagnosis.

In addition, all of the latter mentioned MR-based techniques can be influenced negatively by technical limitations. For example, a small size or irregular shape of the lesion might affect negatively the data analysis. Inappropriate ROI placement (e.g., in heterogeneous lesions with cystic and necrotic regions) and partial volume effects may alter results. Furthermore, susceptibility artifacts due to air-containing and bony structures (e.g., sinuses, temporal bone) adjacent to the lesion may hinder data interpretation and analysis [25,33]. Additionally, for assessment of small lesions (< 2 ml) using MRS, the signal-to-noise ratio becomes a limiting factor requiring examination times inapplicable for clinical use [34]. In a previous study, the rate of unusable MRS examinations due to motion artifacts was about 7% [35].

Regarding the reported patient, susceptibility artifacts and the small lesion size most probably caused the inconclusive findings of PWI MRI and MRS. In contrast, MET PET indicated radiation-induced changes within its limits of the previously described diagnostic accuracy. Ultimately, a stereotactic biopsy demonstrated clearly the absence of vital tumor. Stereotactic biopsy is a highly reliable diagnostic method for differentiating local tumor recurrence from radiation necrosis. Two prospective studies demonstrated the usefulness of this procedure to deliver conclusive results with a high diagnostic accuracy of more than 98% and a low risk (< 1%) of permanent complications [36,37]. Although this procedure is restricted to specialized institutions, stereotactic biopsies can be considered as method of choice, especially when diagnostic information derived from noninvasive imaging techniques are inconclusive.

In conclusion, artifacts and technical limitations of advanced MRI techniques can be a relevant problem and, furthermore, comparative studies are needed to investigate the relationship, diagnostic performance, and complementary character of advanced MRI techniques and amino acid PET. Results of multimodal imaging studies should be evaluated by stereotactic biopsy.

## Consent

Written informed consent was obtained from the patient for publication of this case report and any accompanying images. A copy of the written consent is available for review by the Editor-in-Chief of this journal.

## Competing interests

All authors disclose any financial and non-financial competing interests.

## Authors’ contributions

PK, NG, and MIR participated in the conception, organization and execution of the present work, data analysis and interpretation, and the writing and critical review of the manuscript drafts. FD participated in MRI data acquisition and procession, and MRI data interpretation and the critical review of manuscript drafts. TB participated in the interpretation of neuropathological findings and the critical review of manuscript drafts. MS and NG participated in PET data acquisition and procession, and PET data interpretation and the critical review of manuscript drafts. MK participated in the critical review of manuscript drafts. All authors read and approved the final manuscript.
